# Phylogenetic analysis of the vertebrate Excitatory/Neutral Amino Acid Transporter (SLC1/EAAT) family reveals lineage specific subfamilies

**DOI:** 10.1186/1471-2148-10-117

**Published:** 2010-04-29

**Authors:** Matthias Gesemann, Annegret Lesslauer, Colette M Maurer, Helia B Schönthaler, Stephan CF Neuhauss

**Affiliations:** 1 University of Zurich, Institute of Molecular Life Sciences, Winterthurerstrasse 190, CH-8057 Zurich, Switzerland; 2 Swiss Federal Institute of Technology (ETH) Zurich, Department of Biology, Switzerland; 3 Fondation BBVA-Cancer Cell Biology Programme, Spanish National Cancer Reseach Center (CNIO), E-28029 Madrid, Spain

## Abstract

**Background:**

The composition and expression of vertebrate gene families is shaped by species specific gene loss in combination with a number of gene and genome duplication events (R1, R2 in all vertebrates, R3 in teleosts) and depends on the ecological and evolutionary context. In this study we analyzed the evolutionary history of the solute carrier 1 (SLC1) gene family. These genes are supposed to be under strong selective pressure (purifying selection) due to their important role in the timely removal of glutamate at the synapse.

**Results:**

In a genomic survey where we manually annotated and analyzing sequences from more than 300 SLC1 genes (from more than 40 vertebrate species), we found evidence for an interesting evolutionary history of this gene family. While human and mouse genomes contain 7 SLC1 genes, in prototheria, sauropsida, and amphibia genomes up to 9 and in actinopterygii up to 13 SLC1 genes are present. While some of the additional *slc1 *genes in ray-finned fishes originated from R3, the increased number of SLC1 genes in prototheria, sauropsida, and amphibia genomes originates from specific genes retained in these lineages.

Phylogenetic comparison and microsynteny analyses of the SLC1 genes indicate, that theria genomes evidently lost several SLC1 genes still present in the other lineage. The genes lost in theria group into two new subfamilies of the *slc1 *gene family which we named *slc1a8/eaat6 *and *slc1a9/eaat7*.

**Conclusions:**

The phylogeny of the SLC1/EAAT gene family demonstrates how multiple genome reorganization and duplication events can influence the number of active genes. Inactivation and preservation of specific SLC1 genes led to the complete loss of two subfamilies in extant theria, while other vertebrates have retained at least one member of two newly identified SLC1 subfamilies.

## Background

Genomes of extant species are shaped by extensive gene loss and duplication events. In the radiation of vertebrate species, two whole genome duplication events at the base of the vertebrate lineage (approximately 500 million years (Mya) ago) [1-3] and a third round of genome duplication at the base of the teleost lineage (about 350 Mya ago [4-7]) are proposed to play a crucial role [1-3]. Duplicated genes are expected to be functionally redundant and therefore released of selective pressure, consequently leading to gene loss. Nevertheless many duplicated paralogs are retained in modern genomes, for instance an estimated 15 - 24% of duplicated paralogs are present in extant teleost genomes [6,8-10]. The duplication-complementation-degeneration model seeks to explain the retention of duplicated genes by subfunctionalization, where the function of the essential ancestral gene is distributed to two genes, each fulfilling part of the ancestral gene's function due to regulatory mutations [11,12]. Another most interesting possible event following gene duplication is neofunctionalization, in which one of the two paralogous genes is free to acquire a new function that differs from the ancestral gene, due to the essential (ancestral) function being carried out by the unchanged paralog. Such functionalization events may pave the way for speciation. An alternative driving force in speciation might be divergent resolution, where random paralog losses in two allopatric populations can lead to diversity [13-15]. While whole genome duplications are one way to increase the gene repertoire within a genome, tandem duplication events as well as lineage specific gene loss are other means to change the number of active genes (e.g. [16,17].

In the context of lineage specific gene loss and duplication events, we studied members of the vertebrate *slc1 *gene family of neutral and excitatory amino acid transporters (EAATs), whose presence on glia cells or neurons is indispensable for precise and sustained synaptic activity [18-22]. EAATs are involved in the removal of glutamate from excitatory synapses, a process not only essential to ensure precise termination of synaptic transmission but also to avoid neurotoxic accumulation implicated in a number of diseases [23]. Moreover, transport of glutamate is associated with an increased chloride conductance across the membrane, which hyperpolarizes cells [24]. Based on these important functions high-affinity glutamate transporters in the nervous system are supposed to be under strong purifying selection (pressure to stay the same).

In mammals, five EAAT genes (EAAT 1-5) together with two neutral amino acid transporters form the 'solute carriers 1' (SLC1) gene family (for detailed information on nomenclature, refer to Table 1). In these species EAAT genes have diversified both in spatial and temporal expression and in functional properties and are expressed at glutamatergic synapses throughout the central nervous system. SLC1A3/EAAT1 and SLC1A2/EAAT2 are predominantly expressed in glia cells and presumably mediate the main load of glutamate re-uptake while eliciting only small chloride currents [18,20]. The neuronal transporters, SLC1A1/EAAT3, SLC1A6/EAAT4 and SLC1A7/EAAT5, exhibit larger chloride currents, especially the cerebellar SLC1A6/EAAT4 and the retina-specific SLC1A7/EAAT5 [18,20].

**Table 1:** Gene Aliases in Different Nomenclatures.

SLC1 nomenclature	EAAT nomenclature	Other aliases
SLC1A1	EAAT3	EAAC1
SLC1A2	EAAT2	GLT-1
SLC1A3	EAAT1	GLAST, GLAST-1
SLC1A4		
SLC1A5		
SLC1A6	EAAT4	
SLC1A7	EAAT5	

SLC1 = Solute Carrier Family 1; EAAT = Excitatory Amino Acid Transporter; both nomenclatures originally describe the gene families in man. SLC1A4 and SLC1A5 belong phylogenetically to the SLC1 family, mediate however different functions than EAAT and are therefore missing from the EAAT nomenclature. Data are based on the official HUGO instructions http://www.genenames.org.

In teleosts, given the evidence for a third round of genome duplication (R3), an increased number of SLC1 genes are expected in their genomes. We indeed identified up to 13 SLC1s in fish genomes. While some of these additional SLC1 genes clearly are the consequence of the teleost specific R3, others are evidently members of two additional subfamilies (now called SLC1A8/EAAT6, SLC1A9/EAAT7), that were lost in the lineage leading to human and mouse. Interestingly members of these two subfamilies can also be found in prototherian, sauropsidan, and amphibian genomes, suggesting that these genes are specifically retained in these lineages.

## Results

### The zebrafish genome contains 13 *slc1 *genes on separate chromosomal locations

As a basis to study the evolutionary history of a given gene family, we analyzed the abundance of *slc1 *genes in the zebrafish genome. While in the human and mouse 7 different members of the *slc1 *gene family have been described, we identified and annotated 13 *slc1 *family genes in the zebrafish genome. Cloning from whole embryo cDNA indicated that all of the 13 annotated *slc1 *sequences are indeed transcribed. Sequencing of the amplified cDNA fragments revealed no significant deviation from our predicted sequences, except for some sites of single nucleotide polymorphisms.

The fact that the *slc1 *gene family in zebrafish consists of nearly twice as many genes as present in genomes of human and mouse suggests that at least some of the zebrafish *slc1 *orthologs have originated from the teleost specific whole-genome duplication. Physical and virtual mapping indicated that none of the sequences within a subgroup of *slc1 *are located within the same chromosomal cluster (Additional File 1), supporting the hypothesis that the genes originated from whole genome duplications rather than from individual tandem duplications.

Gene duplicates originating from whole genome duplications should be parts of large blocks of duplicated gene pairs, called paralogons. This holds true for *slc1a3a/eaat1a *and *slc1a3b/eaat1b*, which are located on chromosome 10 and 5 respectively. Moreover, these chromosomes are derived from a common protochromosome, further supporting a whole genome duplication event [7,8].

**Figure 1:**
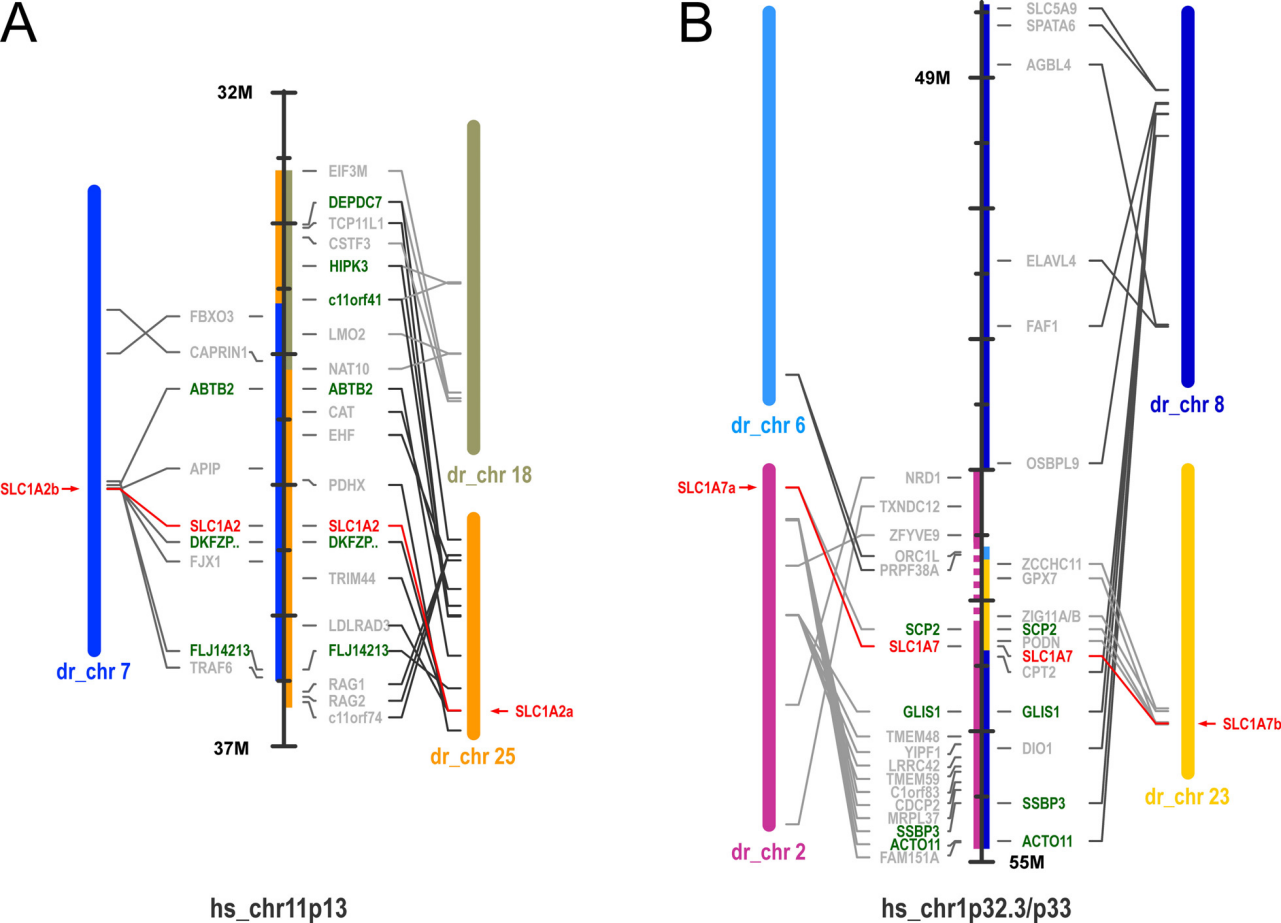
**Synteny of the SLC1A2/SLC1A7 genes**. Genes located within the genomic regions from the human chromosomes 11p13 (A) and 1p32.3/p33 (B) are shown. Synteny between human regions flanking the SLC1A2 and SLC1A7 genes and zebrafish show several genes that are, in addition to the SLC1 genes, duplicated in the zebrafish genome. Note that *slc1a7b *is located on a small orthologous region to the human chromosome 1 covering only 6 genes. Zebrafish chromosomes 7 and 25, harbouring the *slc1a2a *and *slc1a2b *genes, have been previously reported to derive from a common protochromosome [8]. Duplicated genes in the zebrafish genome are shown in green and SLC1 genes are highlighted in red.

The microsynteny analysis of *slc1a2a/eaat2a *and *slc1a2b/eaat2b *revealed that these genes are located within two chromosomal regions on chromosomes 7 and 25 that show multiple duplicated genes (Fig. 1a). These results confirm the assumption that also the chromosomes 7 and 25 are derived from a common protochromosom {Taylor, 2003 #97; Postlethwait, 2000 #208} The microsynteny analysis for the *slc1a7a/eaat5a *and *slc1a7b/eaat5b *genes however gave a more complex pattern. While the *slc1a7b/eaat5b *gene is located on a small chromosomal fragment on chromosome 23 that shows only a handful of genes with conserved synteny to the human chromosome 1, the *slc1a7a/eaat5a *gene is located on chromosome 2 harbouring many genes that have a similar alignment on the human chromosome 1 (Fig. 1b). Strikingly the SCP2 gene, which on the human chromosome is adjacent to the *SLC1A7/EAAT5 *gene can also be found next to *slc1a7a/eaat5a *and *slc1a7b/eaat5b *genes in zebrafish, indicating that this region has been as expected duplicated during evolution.

### Evidence for the existence of additional SLC1 subgroups

**Figure 2:**
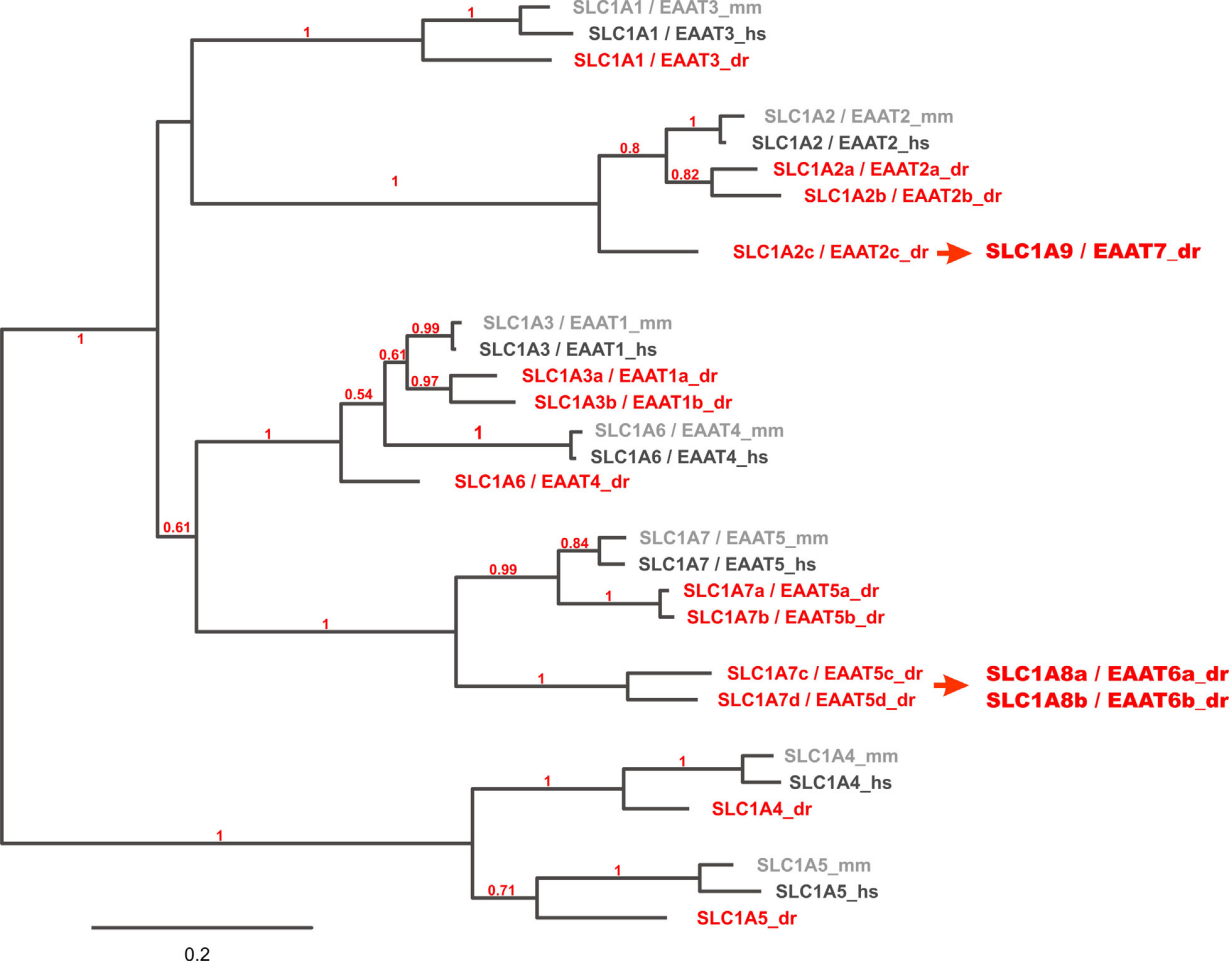
**Phylogenetic analysis of SLC1 genes marks duplication and deletion events**. Maximum likelihood phylogeny of members of the zebrafish (dr), mouse (mm) and human (hs) SLC1 family. The phylogenetic tree was builtusing 370 representative amino acids determined by the program Gblocks after sequence alignment using MUSCLE. Bootstrap values above 50% (0.5) are shown. Zebrafish *slc1 *genes are shown in red. Note that in zebrafish several duplicated *slc1 *genes have been inactivated during evolution, but a duplicated gene for the SLC1A3 family has been retained. Interestingly two subfamilies (*slc1a2 *and *slc1a7*) contain even more than two putative zebrafish orthologs pointing towards the existence of species specific SLC1 subfamilies. The scale bar shows the percentage (0.2 equals 20%) of amino acid substitutions required to generate the corresponding tree. Note that using a Bayesian algorithm to calculate this tree reveals some potential differences in phylogeny (see Additional File 2).

We subsequently analyzed the phylogenetic relationships of the identified zebrafish *slc1 *genes with their corresponding human and mouse orthologs. Interestingly, zebrafish *slc1 *family members do not as expected segregate into 7 clearly distinguishable subgroups with two orthologs each, but rather show a variable number of zebrafish paralogs within the different groups (see Fig. 2 and Additional File 2). While in zebrafish only one ortholog for SLC1A1/EAAT3, SLC1A4, SLC1A5 and SLC1A6/EAAT4 exists, two orthologs for SLC1A3/EAAT1, three orthologs for SLC1A2/EAAT2 and even four genes most closely related to SLC1A7/EAAT5 were found, suggesting that in zebrafish additional *slc1 *subgroups exist.

**Figure 3:**
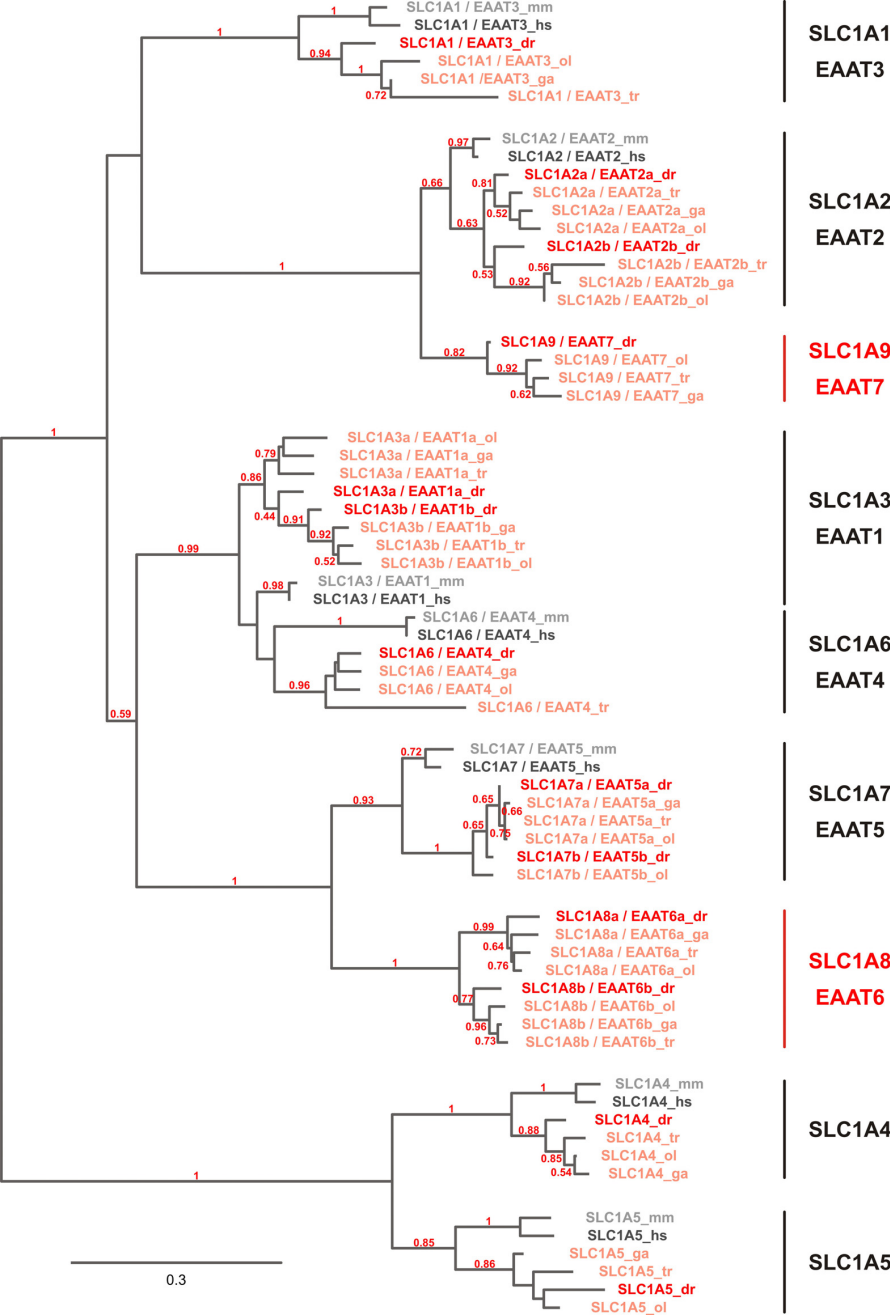
**Teleosts display a conserved number of retained *slc1 *genes**. For this analysis the following species in addition to mouse (mm) and human (hs) were used: Zebrafish *Danio rerio *(dr), *Takifugu rubripes *(tr), Medaka *Oryzias latipes *(ol) and the stickleback *Gasterosteus aculeatus *(ga). The phylogenetic tree was build using the maximum likelihood method on a 331aa stretch determined by the program Gblocks after MUSCLE alignment of the full-length SLC1 amino acid sequence. Bootstrap values above 50% (0.5) are shown. While zebrafish *slc1 *genes are shown in dark red, other teleost *slc1 *genes are highlighted in light red. Within the teleost family the number and phylogeny of *slc1 *genes is highly preserved, with the exception of *slc1a7b/eaat5b *which could neither be found in stickleback nor in torafugu. In case of *slc1a9 *the *takifugu rubripes *gene was replaced by the *Tetraodon *gene as the takifugu *slc1a9 *gene could only be partly assembled. Note that teleost *slc1a2c/eaat2c *and *slc1a7c/d/eaat5c/d *genes are phylogenetically clearly separated from other SLC1 subfamilies pointing towards the existence of two additional species specific subfamilies now called *slc1a8 *and *slc1a9*. The scale bar shows the percentage of amino acid substitutions required to generate the corresponding tree.

Given this unexpected result, we analyzed *slc1 *gene families in other teleost genomes (*Oryzias latipes*, *Takifugu rubripes*, *Gasterosteus aculeatus*). Even teleost genomes only distantly related to zebrafish displayed an almost identical number and phylogeny of *slc1 *family members as those identified in the zebrafish genome (Fig. 3), suggesting that within the teleost lineage this number of *slc1 *family members is common. Moreover, members within the defined subgroups *slc1a2/eaat2 *and *slc1a7/eaat5 *clearly segregate into two separate branches, suggesting that in teleosts two additional *slc1 *subgroups, now named *slc1a8 *and *slc1a9*, exist.

**Figure 4:**
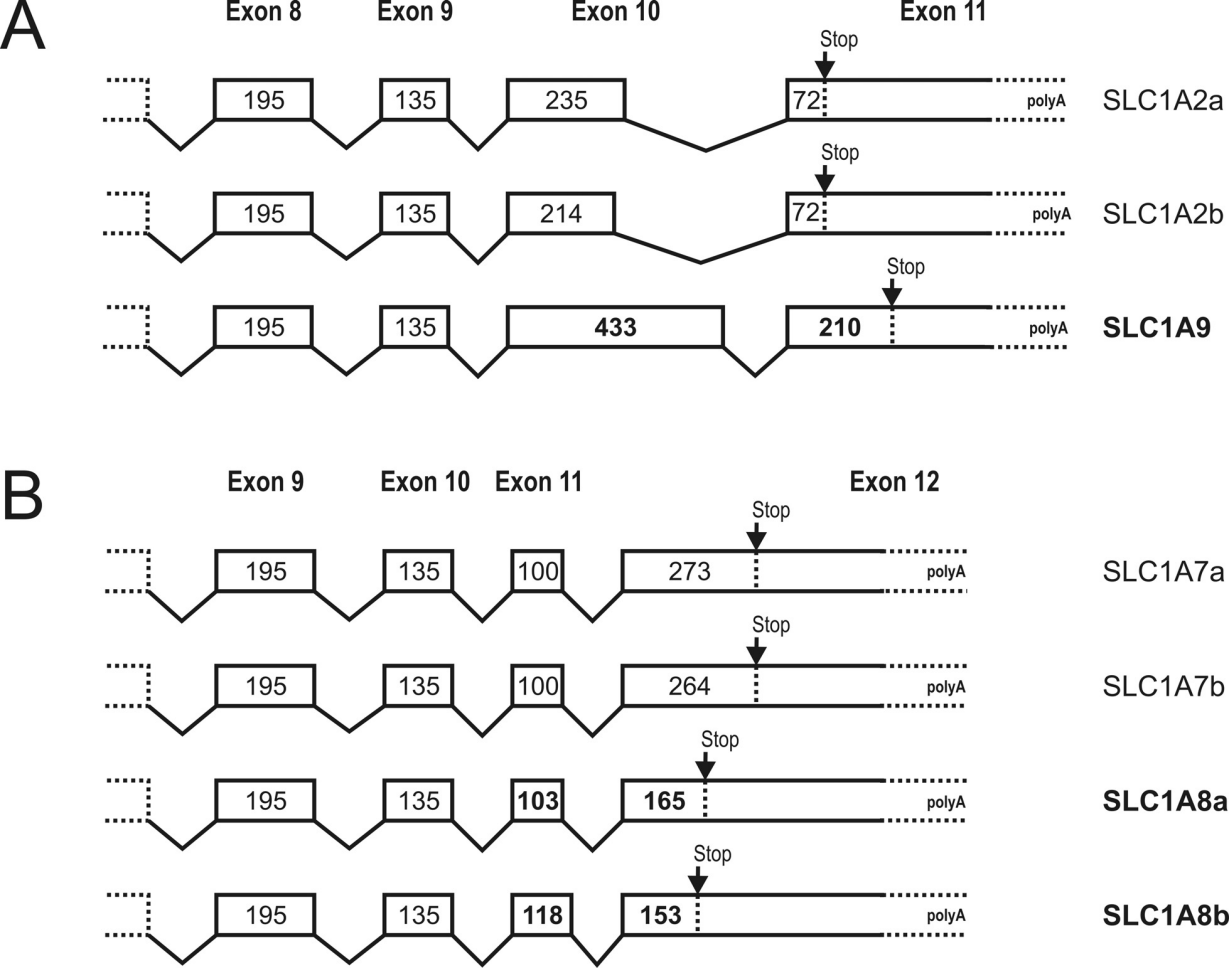
**Members of the new teleost specific SLC1 subfamilies show changes in coding sequence length and exon size**. Analysis of the last two exons within the *slc1a2/9 *(A) and *slc1a7/8 *(B) subfamilies reveals an altered exon size and coding sequence length. While the coding sequence encoded by the last two exons of the *slc1a9 *gene are longer than the corresponding coding sequence of the *slc1a2 *family, exons of the members of the *slc1a8 *subfamily have a shorter coding sequence. Changes within the new subfamilies are highlighted in bold. Note that only *Danio rerio *genes are shown, but that the exon alignment of other teleost fish species gave comparable results.

This assumption was supported by an analysis of the intron/exon structure of the additional subfamilies. While we found a high conservation of the exon number and their corresponding lengths within a given subgroup, the exon size in the newly defined subfamilies were altered (Fig. 4).

### SLC1 gene retention and loss across vertebrate lineages

**Figure 5:**
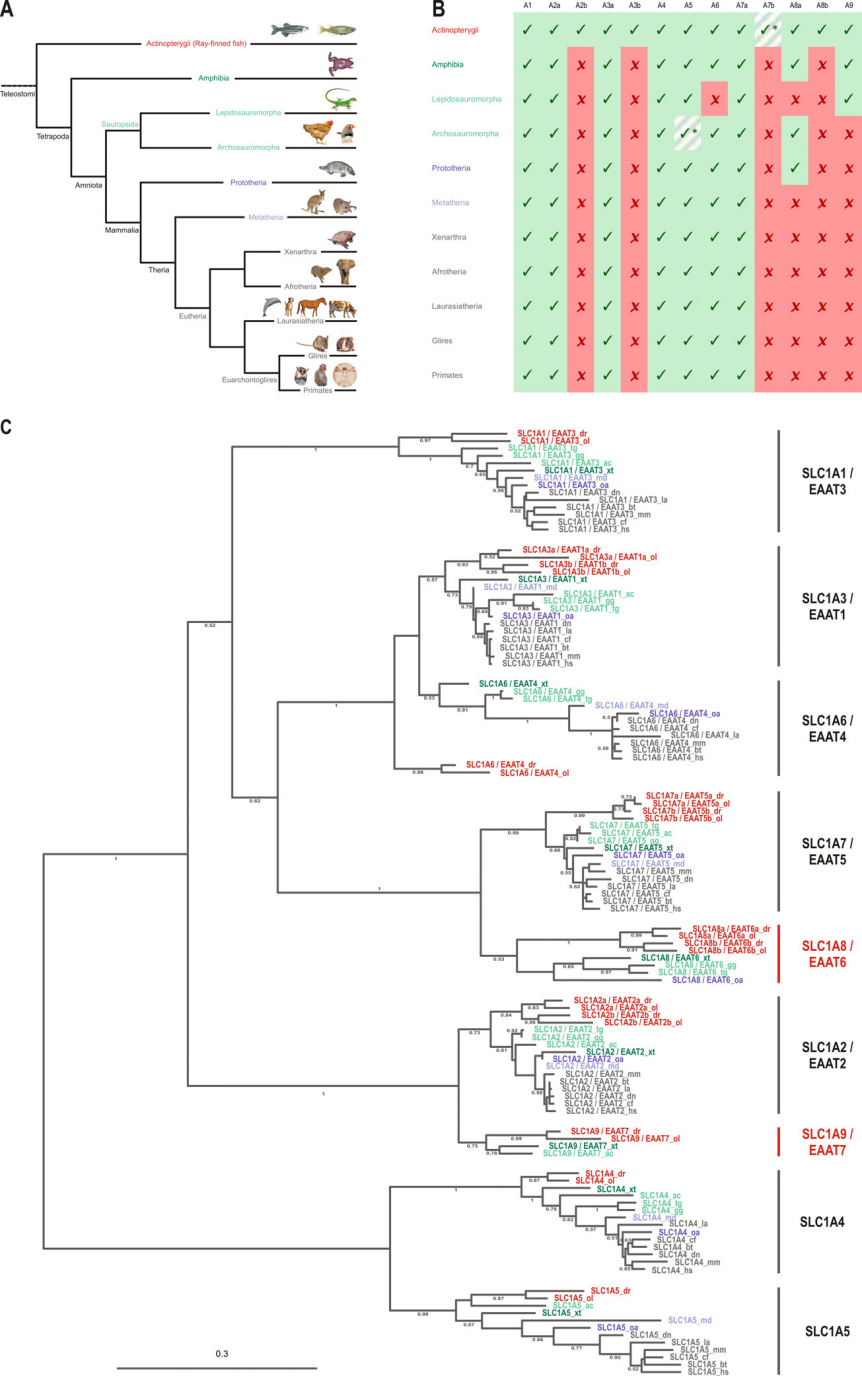
**Selective gene loss within the vertebrate lineage leads to species specific SLC1 subfamilies**. (A) Schematic representation of the vertebrate phylogenetic tree. Major superclasses, classes and infraclasses are indicated and color coded for easier comparison and identification. The species used in the following phylogenetic analysis are shown by representative pictures. (B) The number of active SLC1 genes varies within different species. Due to the fish specific genome duplication (R3), ray finned fish have an increased number of SLC1 genes as indicated by the presence of 'b' paralogs, which cannot be found in other vertebrates. Note that also within the actinopterygian lineage selective gene loss occurs (striped background) as no SLC1A7b paralog can be found in the medaka genome. Within the tetrapod lineage, theria have lost both the SLC1A8 and the SLC1A9 gene, whereas prototheria and archosauromorpha have only lost the SLC1A9 and lepidosauromorpha have lost the SLC1A8 gene. Further gene loss can be observed in some avian species (SLC1A5) and in the green lizard *Anolis carolinensis *(SLC1A6). (C) Maximum likelihood phylogeny of analyzed vertebrate species. The phylogenetic tree was build using 361 representative amino acids residues determined by the program Gblocks after sequence alignment using MUSCLE. Bootstrap values above 50% (0.5) are shown. Species belonging to the different superclasses, classes and infraclasses are highlighted in the same colors as used in Figure 5A. Note that members of the newly identified SLC1A8 and SLC1A9 subgroups all group together forming indeed a separate branch within the SLC1 tree (highlighted in red). The scale bar shows the percentage (0.3 equals 30%) of amino acid substitutions required to generate the corresponding tree.

Since we saw the loss of two SLC1 subfamilies while comparing teleosts and euarchontoglires (rodents and primates), we decided to extend our analysis to encompass the whole vertebrate tree (Fig. 5A; for a complete list of species analyzed see Additional File 3). Therefore we analyzed the genomic information from species, covering the main vertebrate lineages including marsupials, monotremata, sauropsides, and amphibiens (Fig. 5A). While the genomic information from the species used for the following analysis cover the main vertebrate lineages, a complete set of data can be found in Additional File 3 (common and scientific names, abbreviations and genomic coverage), Additional File 4 (links to genomic and transcript information), and Additional File 5 (intron/exon sizes of all species analyzed).

Database analyses indicated that all therian species have lost SLC1A8 and SLC1A9. In contrast, the members of egg laying vertebrates (prototheria, amphibia, and sauropsida) display various gene losses and retentions (Fig. 5B). Monotremates (prototheria) and birds have retained SLC1A8 and lost SLC1A9, whereas the reverse pattern holds true for lepidosauromorphes (lizards) that have retained SLC1A9 and lost SLC1A8. *Xenopus tropicalis*, as a representative of the amphibians on the other hand has retained members of both subfamilies. Interestingly, additional gene losses occurred in a variety of lineages. For instance, chicken (*Gallus gallus*) has lost SLC1A5 and the green anole lizard (*Anolis carolinensis*) has lost SLC1A6 (Fig. 5B).

Phylogenetic reconstruction including now SLC1 sequences from teleosts, amphibia, sauropsida and mammalia clearly indicates that the newly assigned SLC1A8 and SLC1A9 each form a separate clade (Fig. 5C).

In order to get an additional glimpse at the evolutionary history of SLC1 genes, we analyzed the genomes of two basal vertebrates, namely the elephant shark (*Callorhinchus milii*) and the sea lamprey (*Petromyzon marinus*). The elephant shark, belonging to the cartilaginous fish, represents an independent evolutionary branch of the vertebrates, whose genomes are thought to have undergone the same number of genome duplications as the mammalian branch [25]. The low (1.4×) coverage of the elephant shark genome prevented the assembly of complete *slc1 *cDNA sequences. Nevertheless, our comparison revealed only exons for one ortholog per subfamily, with the notable exception of the SLC1A2/A9 subgroup, where we detected a retained SLC1A9 ortholog. Interestingly we also identified two paralogs of the SLC1A2/A9 subgroup in the lamprey, a jawless vertebrate at the base of the vertebrate tree (for a phylogenetic tree see Additional File 6).

### Chromosomal location of putative human *SLC1A8 *and *SLC1A9 *remnants

**Figure 6:**
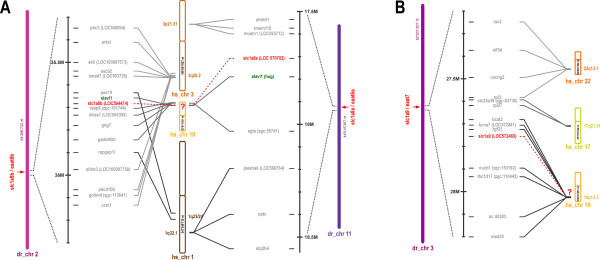
**Synteny of the *slc1a8/slc1a9 *gene family**. Genomic regions around the new zebrafish *slc1a8/slc1a9 *subfamilies are shown. SLC1 genes are highlighted in red. (A) Adjacent to both, the *slc1a8a *and the *slc1a8b *gene respectively, the *elavl1 *gene (highlighted in green) can be found. In addition multiple genes flanking the *slc1a8 *loci localize to the same human chromosomal region (19p13.2/3), (B) Zebrafish genes adjacent to *slc1a9 *can be found on the long arm of the human chromosome 19 in the region q13.3. Note that several genes upstream and downstream of the *slc1a9 *genes are localized within a narrow band (1,6 × 10^6 ^bases) on chromosome 19.

In order to see if remnants of the *SLC1A8 *and *SLC1A9 *genes can still be found within the human genome, we performed a microsynteny analysis localizing genes flanking *SLC1A8 *and *SLC1A9*. Interestingly, zebrafish *slc1a8a *and *slc1a8b *are both flanked by *elavl1*, a gene that can be located to human chromosome 19 (Fig. 6). This human genomic region shows a highly conserved synteny with regions flanking the zebrafish *slc1a8 *genes, suggesting that the inactivated human *SLC1A8 *gene might originally have been located on chromosome 19 in the p13.2/3 region (Fig. 6). Interestingly, the putative inactivated human ortholog of *slc1a9 *has also been located on chromosome 19, however in contrast to *SLC1A8*, which is located on the shorter arm of the chromosome, *SLC1A9 *has been located on the longer arm in the region q13.3. This region also displays a highly conserved synteny, implying that the inactivated gene might indeed have been part of this chromosomal region. However, despite extensive attempts we were not able to identify sequences of the inactivated human *SLC1A8 *and *SLC1A9 *genes, suggesting that the degeneration of these genes has already progressed too far to allow detection.

Furthermore synteny analysis in Xenopus and chicken also revealed ELAVL1 in close proximity to SLC1A8 within the same chromosomal fragment. Similarly we found the NUCB1 gene in the vicinity of SLC1A9 in the Xenopus and Anolis genome (data not shown).

### The expression patterns of zebrafish *slc1a3/eaat1 *and *slc1a2/eaat2 *paralogs suggest subfunctionalization

As an example to compare the spatial and temporal expression pattern of duplicated genes, we investigated the expression patterns of the zebrafish *slc1a3 *and *slc1a2 *subfamily genes.

**Figure 7:**
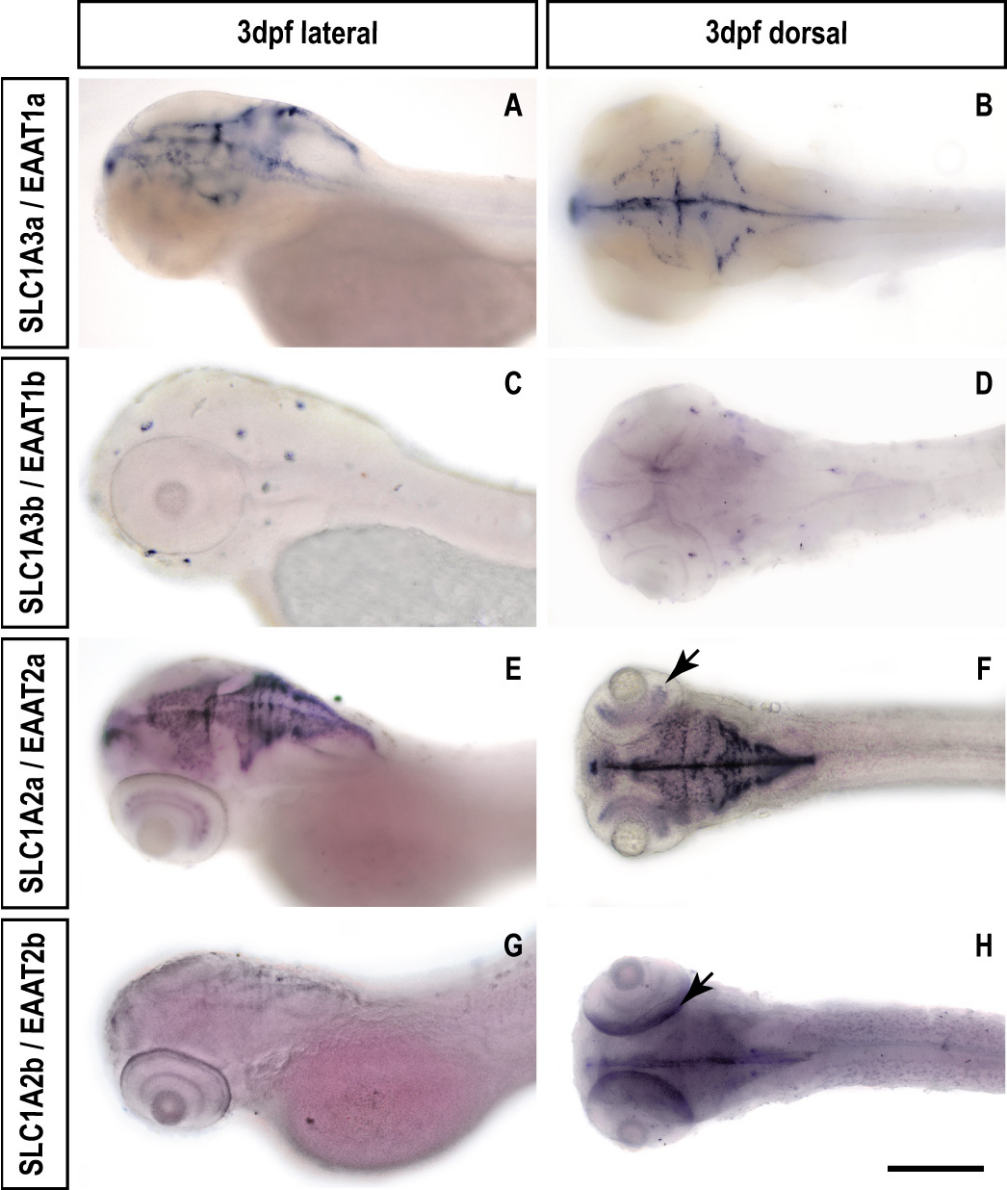
**Zebrafish *slc1a3/eaat1 *and *slc1a2/eaat2 *paralogs show subfunctionalized expression**. The expression patterns of *slc1a3/eaat1 *and *slc1a2/eaat2 *was assessed using whole-mount in situ hybridization on larval (3dpf) zebrafish. While *slc1a3a/eaat1a *shows expression in glia cells of the brain (A and B), *slc1a3b/eaat1b *is expressed in neuromasts of the lateral line organ (C and D). Expression of the *slc1a2 *paralogs overlap in the brain (E-H), however, they differ in the retina. *slc1a2a/eaat2a *is expressed in the inner retina (E and F, arrow), whereas *slc1a2b/eaat2b *is expressed in the outer retina (G and H, arrow). Scale bar is 200 μm.

Whole-mount *in situ *hybridization experiments show that *slc1a3a *transcripts can be found in glia cells of the larval zebrafish brain at 3 days post fertilization (Fig. 7A and 7B), similar to the expression of mammalian *SLC1A3/EAAT1 *in astrocytes of the brain.

We found non-overlapping expression for the *slc1a3b/eaat1b *paralog in neuromasts of the lateral line organ (Fig. 7C and 7D). Neuromasts consist of sensory hair cells and support cells that are homologous to mammalian inner ear hair cells and their adjacent support cells. The observed expression is consistent with a comparable function of this transporter in neuromasts, as observed for the ribbon synapse of the mammalian inner ear [26].

The *slc1a2/eaat2 *paralogs are both expressed in glia cells of the larval zebrafish brain (Fig. 7E-H) in a similar pattern to *slc1a3a/eaat1a*, which is consistent with *SLC1A2/EAAT2 *expression in mammalian astrocytes. In the retina, however, we found a complementary expression pattern with *slc1a2a/eaat2a *transcripts in the inner (Fig. 7E and 7F, arrow) and *slc1a2b/eaat2b *transcripts in the outer retina (Fig. 7H, arrow).

While the expression of zebrafish *slc1a2b/eaat2b *paralogs is in line with mammalian *slc1a2/eaat2 *expression in the outer retina, the *slc1a2a/eaat2a *expression in the inner retina has not been reported in mammals, suggestive of neofunctionalization.

Taken together our expression data for the *slc1a3/eaat1 *and *slc1a2/eaat2 *paralogs reveal subfunctionalization events in the zebrafish, since each paralog fulfills presumably parts of the ancestral function as predicted by the Duplication Degeneration-Complementation model [11,12].

## Discussion

Modern genomes are shaped by frequent duplication and deletion events. Most dramatic are whole genome duplications that were prominently stated by Susumo Ohno as the most important driving force in the evolution of metazoans [1]. This idea has immediate appeal since it provides an elegant route to the generation of new functions without compromising essential ancestral gene functions. Following the split from invertebrates, modern vertebrate genomes harbour remnants of two whole genome duplications [2,3], and there is now overwhelming evidence that ray-finned fishes (teleosts) had an additional whole genome duplication dating back about 320-350 million years ago (Mya) [5], leading to many unique teleost specific genes. While analyzing solute carrier family 1 (*slc1*) genes in zebrafish, we found as expected evidence for many duplicated genes. However, some zebrafish *slc1 *genes turned out to be not the result of the teleost specific R3. Further analyses ruled out the possibility that these SLC1 genes originated from the teleost specific genome duplication. One possible explanation for these additional genes is lineage and species specific gene loss. This has long been recognized in unicellular organisms [27,28]. However more recent reports demonstrate that such events are also observed in vertebrate species [16,29-31], where the impact of such species specific gene loss remains unclear. In the case of the SLC1 family, where five of the seven human and mouse SLC1 genes are grouped in the functionally defined excitatory amino acid transporter (EAAT) family, in teleosts we found members of two additional subfamilies, now called SLC1A8/EAAT6 and SLC1A9/EAAT7, in teleosts. These two subfamilies have been lost in all therian mammals. This is quite surprising, as these proteins are involved in the removal of glutamate from vertebrate synapses and are therefore likely under immediate selective pressure.

In order to reconstruct the phylogenetic history of this gene family we studied a number of vertebrate species from all major vertebrate lineages. We found representatives of both new subfamilies in all analyzed teleost species, showing that the existence of these genes is common to teleost species and is not restricted to zebrafish. Analysis of amphibian genomes reveal the existence of one member of each SLC1A8/EAAT6 and SLC1A9/EAAT7 subfamily, arguing that the tetrapod ancestral species still contained the full complement of SLC1 subfamilies. In this respect it is interesting to note that a study of glutamate transporters in the tiger salamander *Ambystoma tigrinum *retina reports the cloning of a SLC1A8/EAAT6, as well as a SLC1A9/EAAT7 gene (referred to as EAAT2b and EAAT5b in the report, respectively) [32,33], which indeed group into the two new subfamilies (data not shown). These findings provide additional evidence for a scenario where the genes of these subfamilies have been lost after the amphibia - amnionta split.

A clue to when the first subfamily was lost is provided by the sauropsida. Here the Lepidsauromorpha (represented by the green anole lizard *Anolis carolinensis*) have retained SLC1A9 but lost SLC1A8. The situation is reversed in the archosauromorpha (represented by the bird species zebrafinch and chicken), where SLC1A9 was lost but SLC1A8 was retained. This suggests that the genome of the last common ancestor of the sauropsida still contained both subfamilies, followed by independent gene loss in the two branches of the sauropsida. Following this logic the genome of the amniote ancestors should have contained members of both subfamilies.

In mammals only the protherian species (represented by the duck-billed Platypus *Ornithorhynchus anatinus*) have retained the member of SLC1A8, while all other remaining mammals have lost members of both subfamilies. In the most parsimonious scenario the last common mammalian ancestor has already lost SLC1A9 but has retained SLC1A8. Subsequently SLC1A8 was lost at the base of the therian lineage, whereas a member of this subfamily was retained in the branched off protheria species. Hence the SLC1A8 and SLC1A9 subfamilies have been lost independently in a number of lineages. While for instance SLC1A8 has been lost at least twice independently in the archosauromorpha and therian lineage, SLC1A9 has been lost independently in the therian and lepidosauromorpha lineage. In order to infer the ancestral situation we assessed the SLC1/EAAT repository in species that are at the base of the gnathosomes.

Genome information for two species of interest, the elephant shark (*Callorhinchus milii*) and the sea lamprey (*Petromyzon marinus*), is available. While the former represents the cartilaginous fish, an independent branch of the vertebrates that has split before the division of the tetrapod and the teleost lineage, the later belongs to a clade of skulled chordate animals lacking jaws. Since both lineages branched off before R3, cartilaginous fish as well as lampreys are expected to have undergone the same number of genome duplications as the mammalian branch. Consequently the gene repertoire between lamprey, sharks, and mammals should be comparable [25,34]. While we found for each therian SLC1 gene a corresponding elephant shark ortholog, we identified several exons of a retained SLC1A9 ortholog, demonstrating that this basal species have retained members of additional SLC1 gene families. Interestingly we found only one exon sequence for a putative SLC1A8/EAAT6 ortholog in the lamprey and no trace of this gene in the elephant shark. This suggests that either this gene has been lost independently in both of these basal species (assuming that the putative lamprey exon is a false positive one) or that limited genomic information prevented us from identifying these corresponding genes.

Up to now, we have focused our discussion mainly on the SLC1A8 and SLC1A9 subfamilies. An interesting case is also the loss of SLC1A6/EAAT4 in the green anole lizard. This gene is not duplicated in any species analyzed and is prominently expressed in the cerebellum. We can only speculate that another SLC1 gene in lizards must have taken over the function of SLC1A6 in glutamate removal from cerebellar synapses. Support for such a scenario comes from a study comparing the similarity of the glutamatergic sytem in the turtle *Chrysemys picta *and rodents [35].

We have documented multiple gene loss in vertebrate lineages, raising the question if traces of these lost genes can still be found in extant genomes. Most of these gene losses must have happened many Mya ago, so finding defunct gene sequences is rather unlikely. However by microsynteny analyses, we found the putative genomic location of both lost SLC1 genes in the human genome. Intriguingly both lost genes were most likely located on human chromosome 19. While the putative SLC1A8 location is on the short arm of chromosome 19 (19p13.2/3), the most likely position of the inactivated SLC1A9 is on the long arm at position 19q13.3. For this human chromosome extensive rearrangements within the short arm and many intrachromosomal breaks in the long arm have been reported, even since the time of primate rodent divergence [36]. Therefore it seems well conceivable that many genes, including SLC1A8 and SLC1A9, may have been rendered non-functional during these rearrangement events. Our microsynteny analyses indicated that the ELAVL1 gene is in close proximity of both zebrafish SLC1A8 genes, and that other genes in this region map like ELAVL1 to the human chromosome 19. Interestingly this synteny is conserved in chicken and Xenopus, where ELAVL1 and SLC1A8 are neighbors on chromosome 28 (890K-930K) and scaffold 112 (300K-400K) respectively (data not shown).

## Conclusion

The phylogenetic analysis of a gene family spanning multiple taxa can yield important information about their evolutionary history. In the case of the solute carrier family 1, consistent with the duplication-complementation-degeneration model, most of the 7 human or mouse orthologs have one or two teleost orthologs. However, for two teleost SLC1 subfamilies we found no corresponding therian counterpart. This indicates that therians must have lost these two subfamilies, now named SLC1A8/EAAT6 and SLC1A9/EAAT7.

These two subfamilies are not teleost specific, but can be found throughout different vertebrates. An analysis of the major vertebrate lineages revealed an intriguing pattern of lineage specific gene losses, shaping the phylogenetic history of SLC1 genes. In all non-therian vertebrate species we found at least one member of the SLC1A8/9 subfamilies. Amphibians have neither lost SLC1A8 nor SLC1A9, lepidosauria (extent lizards, snakes and tuatara) on the one hand have lost the SLC1A8 ortholog, while birds on the other hand have lost SLC1A9. Interestingly egg laying mammals (platypus) are the only mammals that have also retained a SLC1A8 ortholog.

By studying one gene family across a number of vertebrate taxa, inferences can be made about the evolutionary history of these genes. Such an analysis is needed to guide the interpretation of functional data obtained for those genes and provides a fascinating scenario to study the evolution of vertebrate gene families.

## Methods

### Zebrafish maintenance and breeding

Wild-type fish of the Tübingen (Tü) inbred strain were bred and crossed as previously described [37]. Embryos were raised in E3 medium (5 mM NaCl, 0.17 mM KCl, 0.33 mM CaCl_2_, 0.33 mM MgSO_4_) at 28°C. Stages refer to the development in days post fertilization (dpf).

### Annotation of zebrafish SLC1 cDNAs

As many genes predicted within GenBank are produced by automated processes and have been shown to contain numerous errors such as missing exons and wrong predictions of start and termination sites, all cDNA sequences for the described *slc1 *genes were annotated manually. Sequences from the species listed in Additional File 3 were used for our analysis. Sequences were identified and annotated using combined information from EST, genome (GeneBank, http://www.ncbi.nlm.nih.gov; Ensembl, http://www.ensembl.org/index.html; version 50/51, 2008) and WGS http://blast.ncbi.nlm.nih.gov/Blast.cgi?PAGE=Nucleotides&PROGRAM=blastn&BLAST_SPEC=TraceArchive&BLAST_PROGRAMS=megaBlast&PAGE_TYPE=BlastSearch databases. Human and mouse sequences were used as initial query. Genomic regions coding for SLC1 family genes were identified by using the tblastx alignment algorithm (for details on intron/exon sizes see Additional File 1). Corresponding genomic fragments were analyzed using the GENSCAN gene prediction program http://genes.mit.edu/GENSCAN.html. The obtained putative cDNA and protein sequences were compared to the corresponding human and mouse orthologs and GENSCAN prediction errors were corrected by manual inspection of the intron/exon boundaries in false predicted regions (small exons and minor class splice sites are usually not recognized [38]). Gaps in the assembled sequences due to inaccurate or incomplete genome sequencing were wherever possible filled by corresponding EST or WGS sequences. Sequences found by EST and WGS searches were again blasted against the nr database and only if the top hits corresponded to the original query sequences were used in the annotation process. Note that in the case of duplicated genes annotation errors using WGS sequences could not be excluded due to the fact that individual exons could not be linked together. Sequence alignment of cDNA fragments and overlapping EST sequences was done using the SeqMan software (Lasergene, DNASTAR, Madison WI).

### Phylogenetic tree analysis

Coding sequences of *slc1 *genes (except for the exon 234 analysis which was done on nucleotide sequences) were translated into proteins using the EditSeq software (Lasergene, DNASTAR, Madison WI) and obtained protein sequences were used to generate a combined sequence file in FASTA format (see Additional File 7 (protein FASTA) and Additional File 8 (exon 234 FASTA). Sequence alignment and phylogenetic analysis was performed on the Phylogeny.fr platform http://www.phylogeny.fr/version2_cgi/phylogeny.cgi. Sequences were aligned using MUSCLE (v3.7, Edgar 2004) configured for highest accuracy (MUSCLE with default settings). After alignment, ambiguous regions (i.e. containing gaps and/or are poorly aligned) were removed with Gblocks (v0.91b, [39]) using the following parameters: minimum length of a block after gap cleaning = 10; no gap positions were allowed in the final alignment; all segments with contiguous nonconserved positions bigger than 8 were rejected; minimum number of sequences for a flank position = 85%. The phylogenetic tree was reconstructed using the maximum likelihood method implemented in the PhyML program (v3.0 aLRT, [40]. The default substitution model was selected assuming an estimated proportion of invariant sites and 4 gamma-distributed rate categories to account for rate heterogeneity across sites. The gamma shape parameter was estimated directly from the data. Reliability for internal branch was assessed using the bootstrapping method (100 bootstrap replicates) and bootstrap values above 50% (0.5) are shown. Graphical representation and editing of the phylogenetic tree was done using TreeDyn (v198.3, [41] and the obtained svg files were colored using the CorelDraw program. Animal icons in Figure 5 were purchased from iStockphoto http://www.istockphoto.com.

Alternative alignments and trees were calculated using the alignment programs ClustalW [42] and T-Coffee [43], and the neighbour joining (PHYLIP package 3.66 [44]; distances calculated using ProtDist [45] or MRBAYES (v3.1.2, [46] phylogeny programs. Using these alternative methods revealed some potential differences in phylogeny and a Bayesian calculated tree including human, mouse and zebrafish SLC1 sequences is shown in Additional File 2.

### Cloning

Approximately 50 5 dpf zebrafish larvae were homogenized to prepare total RNA using the Qiagen RNeasy kit (Qiagen), according to the manufacturer's instructions. Oligo dT primers were used to reverse-transcribe the total RNA with reverse transcriptase (First Strand Kit, Invitrogen, Carlsbad, CA). Polymerase chain reaction (PCR) was performed using sequence-specific oligonucleotide primers. Amplified fragments were ligated into the pCR-II vector (TA Cloning Kit Dual Promoter, Invitrogen, Carlsbad, CA, USA) and at least three independently amplified cDNA fragments per gene were sequenced to confirm our previously annotated zebrafish sequences. Accession numbers for the zebrafish SLC1/EAAT genes are: SLC1A1/EAAT3 (HM138690), SLC1A2a/EAAT2a (HM138691), SLC1A2b/EAAT2b (HM138692), SLC1A3a/EAAT1a (HM138693), SLC1A3b/EAAT1b (HM138694), SLC1A4 (HM138695), SLC1A5 (HM138696), SLC1A6/EAAT4 (HM138697), SLC1A7a/EAAT5a (HM138698), SLC1A7b/EAAT5b (HM138699), SLC1A8a/EAAT6a (HM138700), SLC1A8b/EAAT6b (HM138701), SLC1A9/EAAT7 (HM138702).

### Genomic mapping

All zebrafish *slc1 *genes except *slc1a6/eaat4 *were localized in the zebrafish genome by radiation hybrid mapping [47] using the LN54 panel (Loeb/NIH/5000/4000, M. Ekker, Ottawa, Canada) and specific primer pairs for the genomic sequences (see Additional File 9).

### Synteny analysis

For microsynteny analyses, genes flanking the *slc1 *locus of human or zebrafish were used as initial queries for a tblastx [48] search against the zebrafish or human database (ncbi nr/nt database) limited to the desired species (homo sapiens, danio rerio). The different hits were compared with each other and the hit with the highest conservation (identical aa and conserved aa) was selected (note that due to frequently occurring alignment gaps, hits with the lowest expect scores (E values) not necessarily representing the ones with highest identity). Only hits displaying a significant homology in length as well as conservation were used for further analyses. The hit with the highest conservation was used in a reciprocal tblastx search against the corresponding database and only genes which identified the initially used query are depicted.

### Whole-mount *in situ *bybridization

*In vitro *transcription of DIG-labeled probes was performed using the Roche RNA Labeling Kit (Roche Diagnostics, Rotkreuz, Switzerland). RNA probes were hydrolyzed to obtain fragments of about 300 bp length. Whole-mount *in situ *hybridization on 3 dpf zebrafish larvae was performed as follows. PTU treated larvae were anesthetized on ice and immediately fixed in 4% paraformaldehyde in 0.2 M phosphate buffer (pH 7.4) for 40 min at ambient temperature (RT). The *in situ *hybridization was performed according to the Zebrafish Book protocol [50] using an automated *in situ *hybridization machine (BioLane HTI, Hölle & Hüttner, Tübingen, Germany).

## Authors' contributions

MG contributed to cloning, performed the database searches and the phylogenetic analysis, annotated the *slc1 *genes, determined the intron/exon pattern of the *slc1 *genes and performed the synteny analysis. AL cloned the *slc1 *genes used in this study, wrote the first draft of the manuscript and contributed to expression analysis, CMM performed the expression analysis and HBS contributed to cloning and expression analysis. SCFN and MG wrote the manuscript in its present form, and conceived the project and designed experiments. Al authors have read and approved of the final manuscript.

## Supplementary Material

Additional file 1 **Genomic localizations of *slc1 *genes in the zebrafish genome**. Chromosomal localizations were identified both physically, using radiation hybrid mapping, and *in silico*. All zebrafish *slc1 *genes localize to different chromosomal regions. For details on radiation hybrid mapping, see Materials and Methods. *The localization of *slc1a6/eaat4 *could not unambiguously be determined by radiation hybrid mapping and has been mapped *in silico *only. Click here for file

Additional file 2 **Phylogenetic analysis of SLC1 genes using a Bayesian algorithm**. Bayesian phylogeny of members of the zebrafish (dr), mouse (mm) and human (hs) SLC1 family. The phylogenetic tree was build using 370 representative amino acids determined by the program Gblocks after sequence alignment using MUSCLE. The tree was reconstructed using the bayesian inference method implemented in the MrBayes program (v3.1.2). The number of substitution types was fixed to 6. The Poisson model was used for amino acid substitution, while rates variation across sites was fixed to "invgamma". Four Markov Chain Monte Carlo (MCMC) chains were run for 10000 generations, sampling every 10 generations, with the first 250 sampled trees discarded as "burn-in". Finally, a 50% majority rule consensus tree was constructed. Note that in comparison to the maximum likelihood calculated tree, roots in the Bayesian tree are slightly different. While in the Bayesian build tree it appears that there is a common ancestor of SLC1A1, SLC1A2, SLC1A3 and SLC1A6, the tree deriving from maximum likelihood phylogeny suggests that there is a common ancestor between SLC1A3, SLC1A6 and SLC1A7/8 (Figure 2). Zebrafish *slc1/eaat *genes are shown in red. The scale bar represents the percent of amino acid substitutions required to generate the corresponding tree. Click here for file

Additional file 3 **Names, abbreviations and genomic coverage of species analyzed**. The common and the scientific name of the species used are indicated. The genome coverage is given. Note that coverage's highlighted in green (high coverage) and yellow (low coverage) belong to species that have been used to generate the phylogenetic tree covering the major linages. Coverage's shown in red belong to species whose sequences have been annotated but not included in the phylogenetic tree. Click here for file

Additional file 4 **Links to transcript and genome information of the annotated sequences**. The identity of the predicted and the manually annotated sequences is indicated by different colours. Identical predicted and annotated sequences are shown in green, whereas sequences with different predicted open reading frames are highlighted in red. Sequences covering the whole open reading frame are indicated as "Full" sequences and sequences lacking one or more exons are marked as partial sequences. Sequences for which neither a genomic nor a transcript link have been found are given by the letters n.d. (not detected). Click here for file

Additional file 5 **Intron/Exon sizes of SLC1 genes**. The different SLC1 gene families are colour coded. Exons serving as identifiers for the corresponding gene family are highlighted in the identical colour as the gene family. Exons containing the start and the stop codon are highlighted in red. Intron sequences containing multiple place holders (Ns) are indicated. Click here for file

Additional file 6 **The SLC1A9 subfamily is also present in cartilaginous fish**. Database analysis on the elephant shark (*Callorhinchus milii*, cm) and the sea lamprey (*Petromyzon marinus*) genome identified sequences that represent parts of the SLC1A9 gene. For the exon 234 (for more information on *slc1 *intron/exon sizes see supplementary Figure S5), two corresponding *slc1a2 *exons could be found, one of them representing a retained *slc1a9 *gene. The phylogenetic tree was build using the maximum likelihood method on the entire nucleotide sequence of corresponding 234 exons. Bootstrap values above 50% (0.5) are shown. Elephant shark sequences are highlighted in dark red, lamprey sequences are shown in claret red and teleost genes are depicted in light red. Note that the FSGD generated two retained SLC1A2 genes and most likely also two SLC1A9 genes of which in modern teleosts only one is still present. Click here for file

Additional file 7 Protein fastas of species used in the phylogenetic analysis covering the main vertebrate linages. Click here for file

Additional file 8 Nucleotide fastas of the sequences used in the exon 234 analysis. Click here for file

Additional file 9 Oligonucleotide Primers Used for Genomic Mapping. Click here for file
