# Supplementary material for: Phylogenetic analysis of the vertebrate Excitatory/Neutral Amino Acid Transporter (SLC1/EAAT) family reveals lineage specific subfamilies
Source: BMC Evol Biol. 2010 Apr 29;10:117. doi: 10.1186/1471-2148-10-117 (PMC2873418; doi:10.1186/1471-2148-10-117)
Supplement: Additional file 1 — Genomic localizations of slc1 genes in the zebrafish genome. Chromosomal localizations were identified both physically, using radiation hybrid mapping, and in silico. All zebrafish slc1 genes localize to different chromosomal regions. For details on radiation hybrid mapping, see Materials and Methods. *The localization of slc1a6/eaat4 could not unambiguously be determined by radiation hybrid mapping and has been mapped in silico only. [file 1471-2148-10-117-S1.DOC]

**Gene Chromosomal location**

*slc1a1 / eaat3* 7 (70238K – 70262K)

*slc1a2a / eaat2a* 25 (29365K – 29376K)

*slc1a2b / eaat2b* 7 (45447K – 45477K)

*slc1a3a / eaat1a* 5 (66555K – 66610K)

*slc1a3b / eaat1b* 10 (34081K – 34115K)

*slc1a4* 13 (24651K – 24666K)

*slc1a5* 15 (14698K – 14722K)

*slc1a6 / eaat4* 11 (4856K – 4879K)

*slc1a7a / eaat5a* 2 (2674K – 2726K)

*slc1a7b / eaat5b* 23 (38740K – 38893K)

*slc1a8a / eaat6a* 11 (17705K – 17728K)

*slc1a8b / eaat6b* 2 (35676K – 35690K)

*slc1a9 / eaat7* 3 (27724K – 27751K)
