# Supplementary figures and images for: Phylogenetic analysis of the vertebrate Excitatory/Neutral Amino Acid Transporter (SLC1/EAAT) family reveals lineage specific subfamilies
Source: BMC Evol Biol. 2010 Apr 29;10:117. doi: 10.1186/1471-2148-10-117 (PMC2873418; doi:10.1186/1471-2148-10-117)

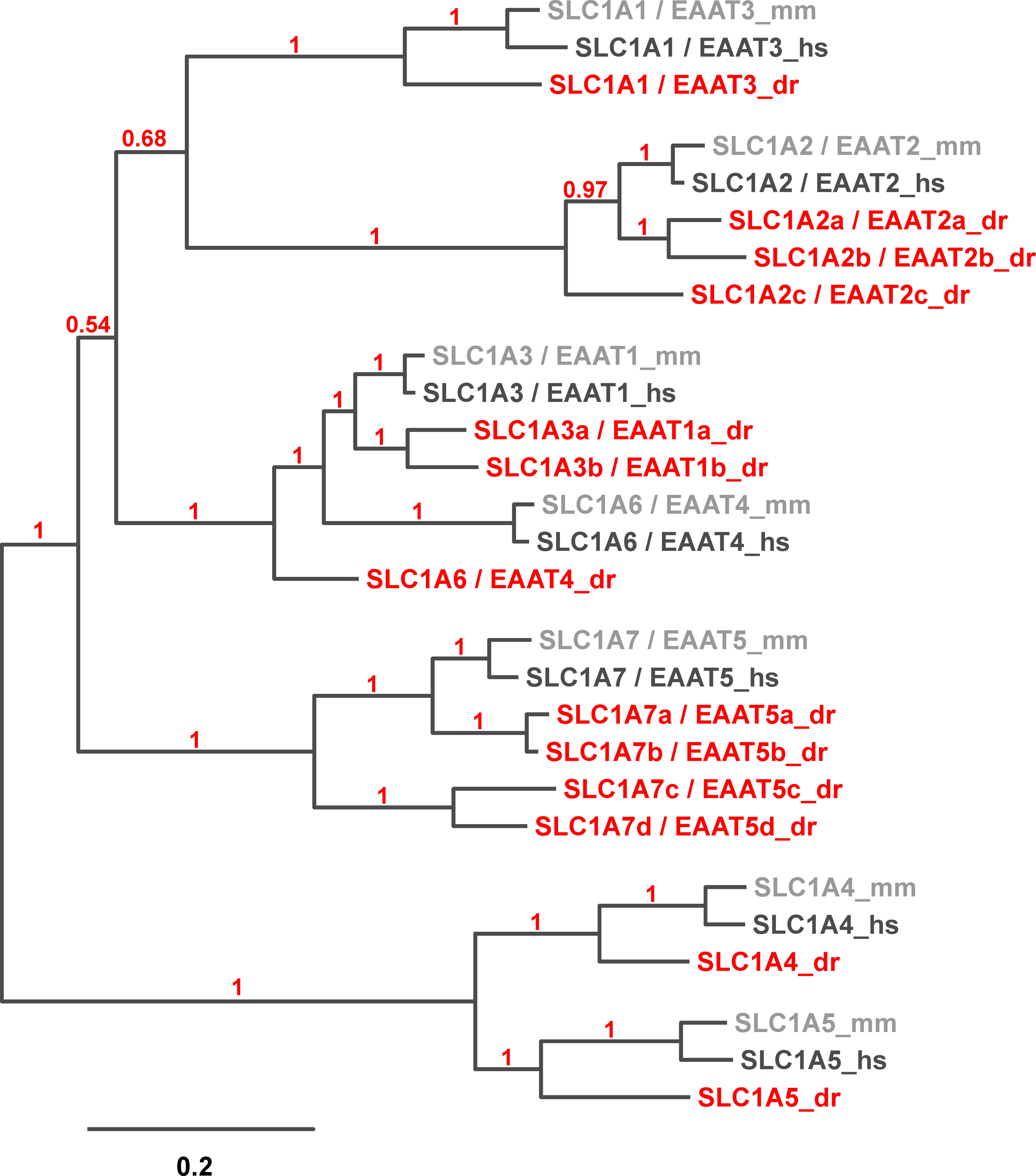

Supplement: Additional file 2 — Phylogenetic analysis of SLC1 genes using a Bayesian algorithm. Bayesian phylogeny of members of the zebrafish (dr), mouse (mm) and human (hs) SLC1 family. The phylogenetic tree was build using 370 representative amino acids determined by the program Gblocks after sequence alignment using MUSCLE. The tree was reconstructed using the bayesian inference method implemented in the MrBayes program (v3.1.2). The number of substitution types was fixed to 6. The Poisson model was used for amino acid substitution, while rates variation across sites was fixed to "invgamma". Four Markov Chain Monte Carlo (MCMC) chains were run for 10000 generations, sampling every 10 generations, with the first 250 sampled trees discarded as "burn-in". Finally, a 50% majority rule consensus tree was constructed. Note that in comparison to the maximum likelihood calculated tree, roots in the Bayesian tree are slightly different. While in the Bayesian build tree it appears that there is a common ancestor of SLC1A1, SLC1A2, SLC1A3 and SLC1A6, the tree deriving from maximum likelihood phylogeny suggests that there is a common ancestor between SLC1A3, SLC1A6 and SLC1A7/8 (Figure 2). Zebrafish slc1/eaat genes are shown in red. The scale bar represents the percent of amino acid substitutions required to generate the corresponding tree. [file 1471-2148-10-117-S2.JPEG]

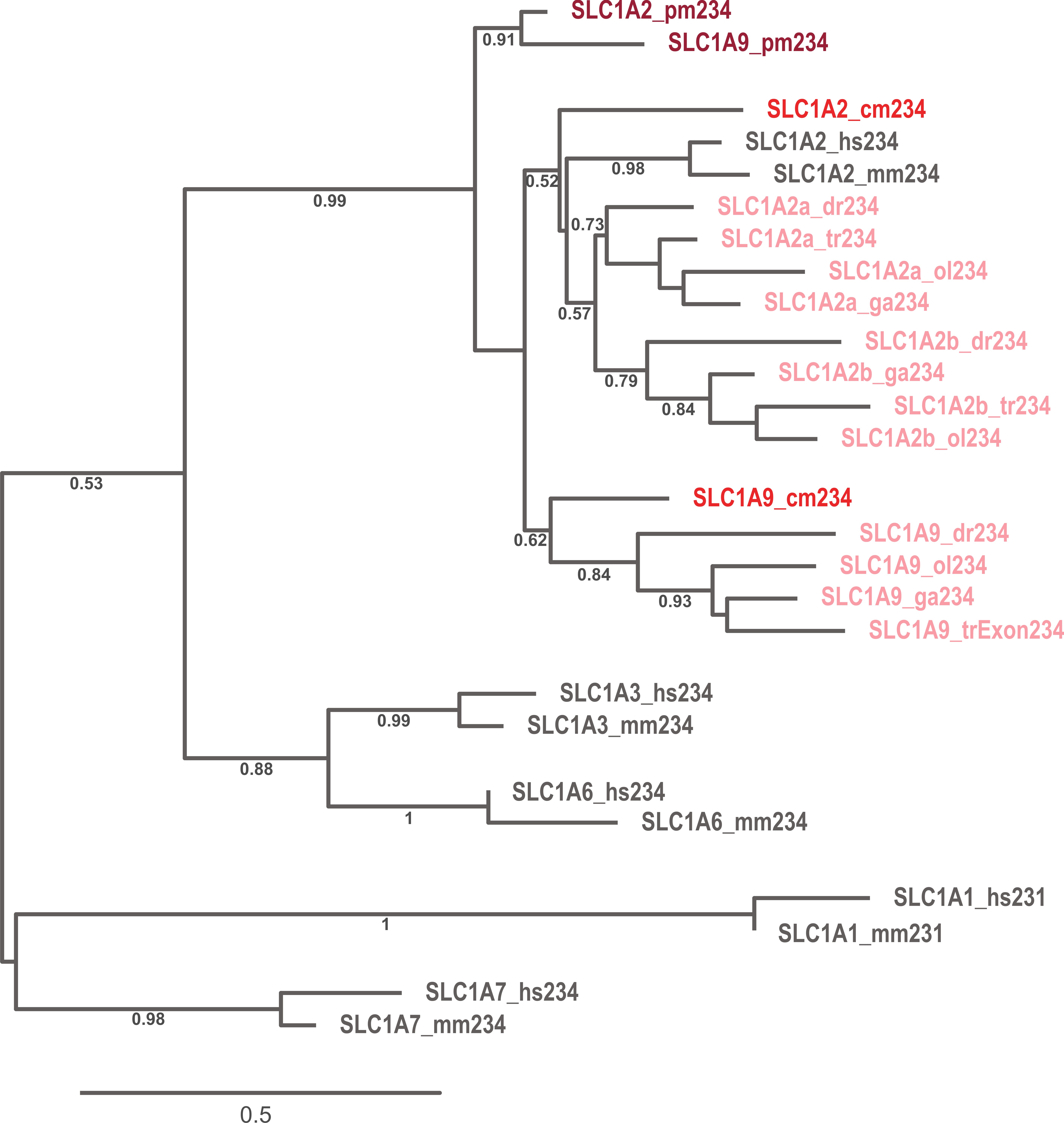

Supplement: Additional file 6 — The SLC1A9 subfamily is also present in cartilaginous fish. Database analysis on the elephant shark (Callorhinchus milii, cm) and the sea lamprey (Petromyzon marinus) genome identified sequences that represent parts of the SLC1A9 gene. For the exon 234 (for more information on slc1 intron/exon sizes see supplementary Figure S5), two corresponding slc1a2 exons could be found, one of them representing a retained slc1a9 gene. The phylogenetic tree was build using the maximum likelihood method on the entire nucleotide sequence of corresponding 234 exons. Bootstrap values above 50% (0.5) are shown. Elephant shark sequences are highlighted in dark red, lamprey sequences are shown in claret red and teleost genes are depicted in light red. Note that the FSGD generated two retained SLC1A2 genes and most likely also two SLC1A9 genes of which in modern teleosts only one is still present. [file 1471-2148-10-117-S6.JPEG]
