# Supplementary material for: Phylogenetic analysis of the vertebrate Excitatory/Neutral Amino Acid Transporter (SLC1/EAAT) family reveals lineage specific subfamilies
Source: BMC Evol Biol. 2010 Apr 29;10:117. doi: 10.1186/1471-2148-10-117 (PMC2873418; doi:10.1186/1471-2148-10-117)
Supplement: Additional file 3 — Names, abbreviations and genomic coverage of species analyzed. The common and the scientific name of the species used are indicated. The genome coverage is given. Note that coverage's highlighted in green (high coverage) and yellow (low coverage) belong to species that have been used to generate the phylogenetic tree covering the major linages. Coverage's shown in red belong to species whose sequences have been annotated but not included in the phylogenetic tree. [file 1471-2148-10-117-S3.DOC]

**Species (common name) Scientific name Abbreviation Genome coverage EST Sequences**

**Mammals**

Human *Homo sapiens* hs Full 8’296’272

Chimpanzee *Pan troglodytes* pt 6 x 5’065

Gorilla *Gorilla gorilla* gog 2 x -

Bornean orangutan *Pongo pygmaeus* pp 6 x -

Rhesus monkey *Macaca mulatta* mam 6 x 58’412

Small-eared galago *Otolemur garnettii* og 1.5 x -

Gray mouse lemur *Microcebus murinus* mim 1.93 x -

Norway rat *Rattus norvegicus* rn Full 951’258

House mouse *Mus musculus* mm Full 4’852’072

Rabbit *Oryctolagus cuniculus* oc 2 x 34’938

Domestic guinea pig *Cavia porcellus* cp 6.79 x 19’975

American pika *Ochotona princeps* op 1.93 x -

Northern tree shrew *Tupaia belangeri* tb 2 x 2’316

African savanna elephant *Loxodonta Africana* la 2 x -

Cape rock hyrax *Procavia capensis* pc 2.19 x -

Small Madagascar hedgehog *Echinops telfairi* et 2 x -

Alpaca *Vicugna pacos* vp 2.51 x 7’286

Horse *Equus caballus* ec 6.79 x 36’937

Cattle *Bos taurus* bt 7 x 1‘517‘145

Pig *Sus scrofa* ss ?? 1‘536‘375

Dog *Canis familiaris* cf 7.5 x 365’909

Domestic cat *Felis catus* fc 1.87 x 919

Western European hegehog *Erinaceus europaeus* ee 1.86 x -

Little brown bat *Myotis lucifugus* ml 1.7 x -

Large flying fox *Pteropus vampyrus* pv 2.63 x -

Bottlenosed dolphin *Tursiops truncatus* tt 2.59 x 2’285

Nine-banded armadillo *Dasypus novemcinctus* dn 2 x -

Hoffman’s two-fingered sloth *Choloepus hoffmanni* ch 2.05 x -

Gray short-tailed opossum *Monodelphis domestica* md 7.33 x 265

Tammar wallaby *Macropus eugenii* me 2 x 14’878

Platypus *Ornithorhynchus anatinus* oa 6 x 9’699

**Species (common name) Scientific name Abbreviation Genome coverage EST Sequences**

**Vertebrates**

Chicken *Gallus gallus* gg 7.1 x 600‘075

Zebra finch *Taeniopygia guttata* tg 6 x 91’801

Western clawed frog *Xenopus tropicalis* xt 7.65 x 1’271’375

Green anole Anolis carolinensis ac 6.3 x 152’802

Zebrafish *Danio rerio* dr 7 x 1’481’930

Torafugu *Takifugu rubripes* tf 8.7 x 26’069

Medaka *Oryzias latipes* ol 10.6 x 665’382

Three spined stickleback *Gasterosteus aculeatus* ga 11 x 276’992

Ghost (Elephant) shark *Callorhinchus milii* cm 1.4 x -

Sea lamprey *Petromyzon marinus* pm 5.9 x 120’731

Top of Form

Bottom of Form
