# Supplementary material for: Phylogenetic analysis of the vertebrate Excitatory/Neutral Amino Acid Transporter (SLC1/EAAT) family reveals lineage specific subfamilies
Source: BMC Evol Biol. 2010 Apr 29;10:117. doi: 10.1186/1471-2148-10-117 (PMC2873418; doi:10.1186/1471-2148-10-117)
Supplement: Additional file 9 — Oligonucleotide Primers Used for Genomic Mapping. [file 1471-2148-10-117-S9.DOC]

| **S9**  Oligonucleotide Primers Used for Genomic Mapping | | |
| --- | --- | --- |
| Gene | Primer | 5’ to 3’ |
| *slc1a3a* | EAAT-1a_dr_104460asgen | TGGCCGTGATGCTACATTAATAATAC |
| *slc1a3a* | EAAT-1a_dr_104144sgen | GTGACAACACTGGCTCTTCATCTATG |
| *slc1a3b* | EAAT-1b_dr_54390asgen | AAACCAATCAACAGCGATAATCAGAG |
| *slc1a3b* | EAAT-1b_dr_54119sgen | CATGGATGCAGGATGTTCTCTTTC |
| *slc1a2a* | EAAT-2a_dr_23017asgen | CTGGCGAGTGTTGCTGTAAGACTAG |
| *slc1a2a* | EAAT-2a_dr_22707sgen | ATCGGTGCTAGTTTTGGAAGTTTATC |
| *slc1a2b* | EAAT-2b_dr_21993asgen | GATCAGAAGCATTGTCACCAAACC |
| *slc1a2b* | EAAT-2b_dr_21693sgen | CAGTTTGTTGAGCTGTGTAAAGATAAAC |
| *slc1a9* | EAAT-7_dr_72655asgen | ACACTGGCTAAAGTCGCTGTCATAC |
| *slc1a9* | EAAT-7_dr_72372sgen | CAGTAAACATCCCTTAAATCTGATTGG |
| *slc1a1* | EAAT-3_dr_42595asgen | AAGCTACTTAAGTGCATTACATCACAGC |
| *slc1a1* | EAAT-3_dr_42298sgen | AAAGGCAATCACACAAAAACTGAAAG |
| *slc1a6* | EAAT-4_dr_70911sgen | CAACGACATGGATCTGAACTTTGG |
| *slc1a6* | EAAT-4_dr_71252asgen | CTATTGGACACAGGTCAGGACTGC |
| *slc1a7a* | EAAT-5a_dr_105384sgen | CAACAACTACGAGCTGGACTTTGG |
| *slc1a7a* | EAAT-5a_dr_105745asgen | ACTAATGATCAGGGGGGATTGTTG |
| *slc1a7b* | EAAT-5b_dr_441043sgen | GACTTCGGCCAAATCATCACAATC |
| *slc1a7b* | EAAT-5b_dr_441419asgen | AATTCAGCATGAGAAAAAGCCTTTG |
| *slc1a8a* | EAAT-6a_dr_78203sgen | TTCAGCAACACTTCCCATTACAATG |
| *slc1a8a* | EAAT-6a_dr_78511asgen | GTAATGCATTCTCCCTTCAATATACTG |
| *slc1a8* | EAAT-6b_dr_57084sgen | AACGAGTATGAGCTTGACTTTGGAC |
| *slc1a8b* | EAAT-6b_dr_57387asgen | GTATCAAAACCATTCATCAAAGTTGTTC |
| *slc1a4* | SLC1A4_dr_80312sgen | GTGTGTTGCTGCTGTGTTTATTGC |
| *slc1a4* | SLC1A4_dr_80736asgen | CAATAGCGTTCCAAAAACTAGTACACC |
| *slc1a5* | SLC1A5_dr_66242asgen | CGTTCAGCTGAGCGATAAACACAG |
| *slc1a5* | SLC1A5_dr_65904sgen | GGCCGAACATGTATTTCCAATTAGC |
